# The Neural Basis of Changing Social Norms through Persuasion

**DOI:** 10.1038/s41598-017-16572-2

**Published:** 2017-11-24

**Authors:** Yukihito Yomogida, Madoka Matsumoto, Ryuta Aoki, Ayaka Sugiura, Adam N. Phillips, Kenji Matsumoto

**Affiliations:** 1 Brain Science Institute, Tamagawa University, Tokyo, 194-8610 Japan; 2 Department of Mental Disorder Research, National Institute of Neuroscience, National Center of Neurology and Psychiatry, Tokyo, 187-8502 Japan; 3 Japan Society for the Promotion of Science, Tokyo, 102-0083 Japan; 4 Department of Life Sci, GSAS, University of Tokyo, Tokyo, 153-8902 Japan

## Abstract

Social norms regulate behavior, and changes in norms have a great impact on society. In most modern societies, norms change through interpersonal communication and persuasive messages found in media. Here, we examined the neural basis of persuasion-induced changes in attitude toward and away from norms using fMRI. We measured brain activity while human participants were exposed to persuasive messages directed toward specific norms. Persuasion directed toward social norms specifically activated a set of brain regions including temporal poles, temporo-parietal junction, and medial prefrontal cortex. Beyond these regions, when successful, persuasion away from an accepted norm specifically recruited the left middle temporal and supramarginal gyri. Furthermore, in combination with data from a separate attitude-rating task, we found that left supramarginal gyrus activity represented participant attitude toward norms and tracked the persuasion-induced attitude changes that were away from agreement.

## Introduction

Social norms are the rules governing acceptable behavior within a group^1–3^. They regulate individual behavior as internalized beliefs and through external interpersonal sanctions, and they regulate important social phenomena such as cooperation, collective action, and social order^2,4,5^.

Importantly, norms are not fixed but dynamically changing within a society^3,6^. These changes can occur because factors such as the natural environment, group composition, or economic systems fluctuate^7,8^. In modern societies, persuasive communication plays an important role in how norms change. While virtually all people agree on some norms such as ‘murder is wrong’, agreement with many other norms varies person to person. When questioning a norm gains significant traction within a society, proponents and critics often attempt to sway others to their respective positions with personal dialog, through television, and over the internet^2,6,9,10^. Indeed, verbal persuasion has been a factor in the abolition of racial segregation through the civil-rights movement, new attitudes against public smoking, and the ongoing changes across some parts of the world with regard to same-sex marriage^7,11,12^.

How much people agree with a norm can be influenced by persuasion, and this process is critical to group-level reinforcement/emergence or weakening/abolition of norms. Recent studies in social science have begun to investigate how this process unfolds in the context of communication in social networks^7,13^, but a biological explanation at the individual brain level is still lacking.

Recent studies have assessed various norm-related cognitive processes and clarified their neural correlates. Norm compliance/adaptation has been associated with the right dorsolateral prefrontal cortex (dLPFC), ventromedial prefrontal cortex (vMPFC), and insula^14–16^. Moral judgment has been associated with the dorsomedial prefrontal cortex (dMPFC), posterior cingulate cortex, the temporo-parietal junction (TPJ), vMPFC, and the striatum^17–19^. Additionally, recognition of social equality has been associated with the activity in the vMPFC, striatum, and insula^20,21^. However, these studies presupposed fixed norms/moral-standards, and therefore did not assess how norms themselves ‘change’. Other recent studies have assessed the neural basis of persuasion in the context of product advertisement or health promotion^22–27^. These studies showed that the dMPFC, vMPFC, inferior frontal gyrus (IFG) and dLPFC play a pivotal role in attitude change through persuasion. However, social norms were not targeted, and whether changing attitudes toward social norms requires any additional brain regions is unknown. Thus, neuroscience studies of social norms have never assessed how norms *change*, while those of persuasion-induced attitude change have not targeted norms.

In the present study, we aimed to clarify the neural substrates critical for attitude change regarding social norms. Specifically, we tested the following three hypotheses: (1) Persuasion targeting norms recruits different brain regions than persuasion targeting non-normative issues. Unlike non-normative beliefs, normative beliefs are more stable and resistant to manipulation^28^. This is likely because normative beliefs are maintained in social communities, which might lead to an additional cognitive demand when changing normative beliefs, which would recruit social cognition-related regions (temporal pole, TPJ, and dMPFC) beyond what is recruited for general persuasion that targets both normative and non-normative beliefs (hereafter, ‘beliefs’).

(2) Different neural substrates are recruited depending on the direction of persuasion. Being persuaded to agree less with a norm is psychologically different from the reverse because one might expect significant changes in the relationships with one’s peers (e.g., rejecting a norm can incur social risk). Thus, the underlying neural processes are very likely to be different. Understanding this difference is important because mass persuasion that acts on norms can bring about quite contrasting social changes. (3) Brain regions that represent attitudes for social norms are involved in the norm-persuasion process. This hypothesis is highly reasonable because the link between the persuasion and the attitude change should be essential for the neural basis of any persuasion, although previous neuroscience studies on persuasion never addressed such a link.

Addressing these hypotheses will fill the gap between previous studies of social norms and persuasion, and provide necessary insight for developing of a full theory that explains how norms change in society.

## Materials and Methods

### Task summary

**Figure 1:**
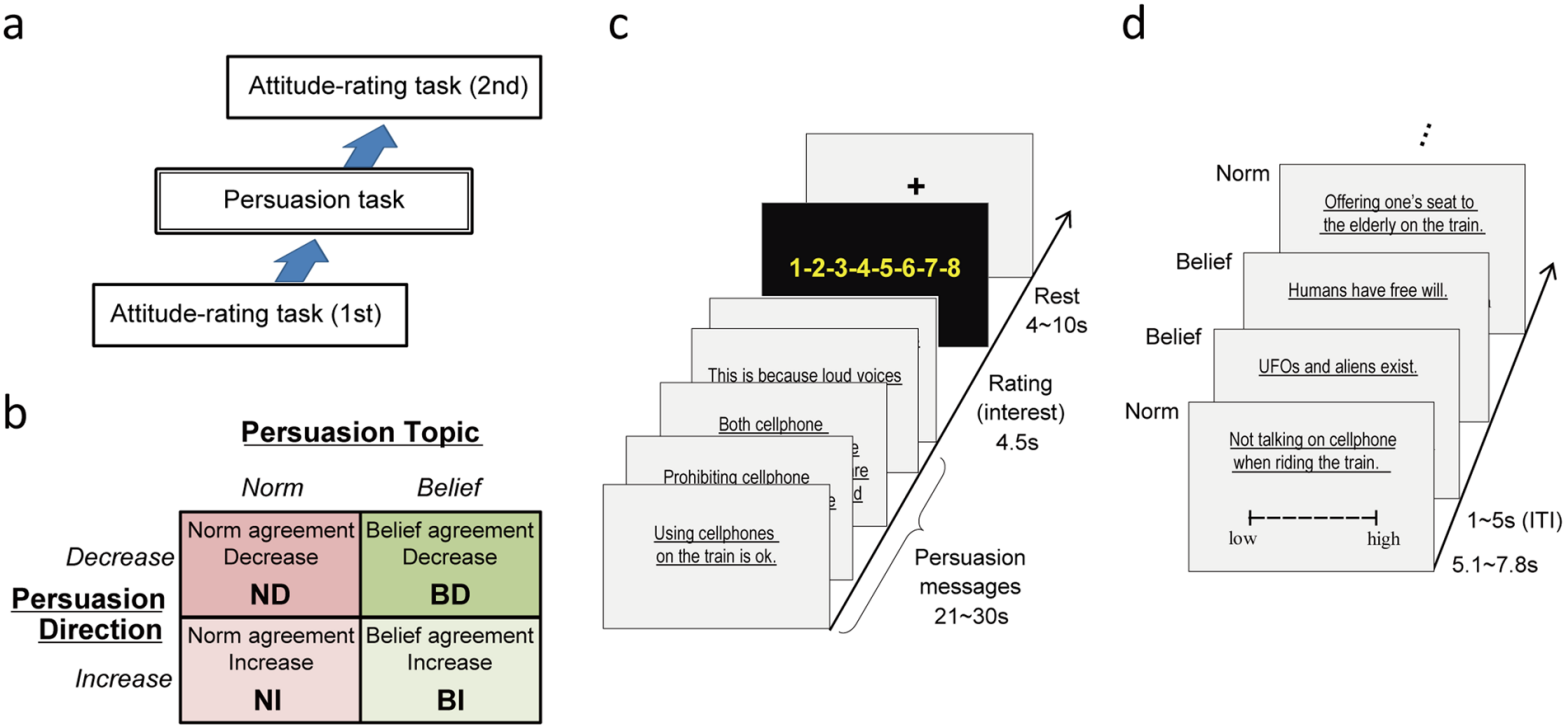
Experimental design. (**a**) Participants rated their attitudes for a wide variety of norms and beliefs that do not address any rules about social behaviors (beliefs) in the first attitude-rating task. They then completed a persuasion task in which messages were designed to change their attitudes about the two norms and two beliefs (the same for each subject and taken from the previous task). Finally, the attitude-rating task was repeated, and any changes in attitudes were revealed. (**b**) The persuasion task had a 2 × 2 factorial design of persuasion topic (norm or belief) × persuasion direction (increasing or decreasing agreement). (**c**) Block structure in the persuasion task. After viewing a persuasive message consisting of five sentences, participant’s rated how interesting they felt the messages were. Each subject completed 24 blocks (6 blocks × 4 conditions: ND, NI, BD, and BI). (**d**) An example sequence of trials in the attitude-rating task. Participants rated how much they agreed with the pseudorandomly presented norms and beliefs. The attitude-rating tasks used 50 norms and 50 beliefs, 4 of which (the same 2 norms and 2 beliefs) were included in the persuasion task.

To test our hypotheses, we recorded brain activity using fMRI while participants underwent a novel persuasion task in which they read four types of persuasive messages. We used a 2 × 2 factorial design in which the factors were “persuasion topic” (norm or belief) and “persuasion direction” (increasing or decreasing agreement) (Fig. 1). We assessed whether norm-targeted persuasion recruits specific brain regions by calculating the main effect of persuasion topic. Interaction effects were assessed to clarify directional specificity in norm-persuasion. Before and after the persuasion task, participants rated their attitudes toward various norms and beliefs, including those that served as the target of persuasion (this was the attitude-rating task). The ratings were used to evaluate each participant’s degree of persuasion-induced attitude change. Additionally, we assessed the correlation between these ratings and brain activity during the attitude-rating task to localize brain regions that represent attitudes for social norms. Subsequently, the localized region’s involvement in the norm-persuasion in persuasion task was assessed.

In addition to the main persuasion experiment described above, we conducted another control experiment with the same design, except that persuasion was absent. This control experiment enabled us to directly compare how attitudes toward the same norms/beliefs change depending on whether persuasion has been used.

### Task and stimuli

#### Persuasion experiment

*Participants*: Twenty-seven right-handed healthy student volunteers (16 females and 11 males; age: 20.81 ± 1.49 years, range: 18–24 years) participated in this experiment. Because most of our analyses dealt with cognitive processes that do not have easy to predict neural correlates (regions), we were not able to employ formal power analysis using *a priori* ROIs or pilot data to determine the sample size^29^. Hence, we chose a reasonable sample size based on related studies^23–25^. All participants were native Japanese speakers who were recruited from the student pool of Tamagawa University and Meiji University (the stimulus preparation and control experiments also used this participant pool). No participant had a history of neurological or psychiatric disease and written informed consent was obtained from all participants prior to participation. The study was approved by the ethical committee of Tamagawa University in accordance with the ethical standards laid down in the 1964 Declaration of Helsinki and its later amendments.

*Procedures*: Participants completed a *persuasion task* and two rounds of *attitude-rating tasks* in the fMRI scanner. They completed the first attitude-rating task, followed by the persuasion task and the second attitude-rating task (Fig. 1a).

### Attitude-rating task

In the 1st-attitude-rating task, participants rated 100 pseudo-randomly presented norms and beliefs (50 norms, 50 beliefs) by indicating how much they agreed with statements regarding each (Fig. 1d). To avoid any nuisance effect caused by positive/negative phrasing, half of the ‘norm’ statements advocated norm compliance (e.g. “Keeping quiet when watching a movie in a theater.”) and the other half advocated prohibition of norm violation (e.g. “Not cutting in line.”). Similarly, statements of belief comprised 25 positively-phrased sentences (e.g. “UFOs and aliens exist.”) and 25 negatively-phrased sentences (e.g. “Petroleum won’t run out within the next fifty years.”). From the 50 norms we chose two norms (Norm_Target1: “Not talking on cellphones when riding the train”; Norm_Target2: “Not reading magazines in convenience stores without buying them”) as the targets for the subsequent persuasion task. Similarity, two of the 50 beliefs (Belief_Target1: “Humans have free will”; Belief_Target2: “Sugar is bad for your health”) were selected as the targets for persuasion. The remaining 48 norms (Norm1–48) and beliefs (Belief1–48) did not undergo persuasion. Among the 50 norms we prepared, four norms explicitly referred to fairness, which has been intensively examined in previous neuroscience norm literature^14,16^. Presentation duration (5,080–7,780 ms) for each statement was adjusted based on the number of characters, and inter-stimulus intervals were jittered between 1,000 and 5,000 ms (average 3,000 ms). Because participants may be reluctant to disclose their true attitudes toward norms due to social desirability^30^, we used a ‘bogus pipeline’ procedure that reduces this bias and improves truthfulness of self-reports^30–32^. We told participants that we were testing their honesty, and presented them with our ‘lie-detector’ (actually an MRI-compatible pulse oximetory sensor). The sensor was attached to their index fingers during scanning, and they were instructed to report how much they agreed with the norms/beliefs by using an eight-point scale (1 = low agreement, 8 = high agreement). Practically, attitude rating was accomplished using two 4-button MRI-compatible button boxes, one for each hand. As each box invariably represented either high or low ratings, the hands (left or right) allocated to each box were balanced across participants so as to separate brain activity related to ratings from that related to motor control.

Participants performed the second attitude-rating task after they completed the persuasion task. The task protocol was exactly the same as the first rating task, except that the pseudo-random order of the norm/belief statements was different.

### Attitude rating scale

The lowest value on the rating scale indicated the null agreement or neutral position toward norms (beliefs), rather than active objection to them. The rationale behind this unilateral scale was as follows. We planned to identify brain regions that represent attitudes toward norms (beliefs) by assessing the correlation between brain activity during attitude-rating tasks and the ratings themselves. This analysis presupposes that the scale measures a qualitatively uniform factor. We felt that this assumption could be undermined if we employed a scale that ranged from ‘complete disagreement’ to ‘complete agreement’. While higher values on this scale pick up positive attitudes that constitute agreement (i.e., praise, support etc.), lower values pick up qualitatively different negative attitudes (i.e., hate, objection etc.), as suggested by previous social psychology^33^ and neuroscience^34,35^ studies. Such a mixture of different factors in a scale might have confounded the simple correlation analysis between ratings and brain activity. Hence, to avoid this problem we limited our scale’s range to reflect a qualitatively uniform factor, agreement. Persuasion thus increased or decreased the magnitude of agreement. For the same reason, in addition to employing a unilateral scale, we only presented norms describing desirable behavior (i.e., injunctive norms) and avoided presenting negative/unpopular norms that people tend to follow but privately do not like (e.g., always agree with ones’ superiors)^36^ in the attitude-rating task. We did so because presenting negative norms could induce negative attitudes and confounded our analysis.

### Persuasion task

In the persuasion task, participants read persuasive messages in the scanner. Because we did not want participants to know our intent was to study persuasion, we told them that the task was a ‘trivia task’ mimicking a famous Japanese TV program (“Trivia-no-izumi”: The Fountain of Trivia). Participants were asked to read trivia presented on the screen, and rate how interesting they were. In reality, four types of persuasive messages were presented in a two-by-two factorial design (Fig. 1b,c). Two types of messages persuaded participants to either increase or decrease their levels of agreement with the norms (Norm agreement Increase [NI] and Norm agreement Decrease [ND], respectively), and two types were control messages designed to either increase or decrease levels of agreement with the beliefs (Belief agreement Increase [BI] and Belief agreement Decrease [BD], respectively). One complete message consisted of five statements that were presented as a block. The first statement expressed the desired conclusion, and the following four statements provided evidence or claims that supported the first statement (the persuasive argument). Example persuasion messages are shown in Table 1.

**Table 1:** Example set of stimuli presented in the persuasion task (translated from the original Japanese).

**[Norm agreement Decrease]**
Using cell phones on the train is ok.
Prohibiting cellphone conversations but not those with people around you is unreasonable.
Both cellphone conversations and those with people around you are prohibited in theaters and libraries.
This is because loud voices are annoying in those situations.
Since normal conversations are not prohibited, cellphones use cannot be deemed annoying.
**[Norm agreement Increase]**
Reading magazines in convenience stores without buying them is bad.
Such behavior reduces the number of magazines purchased, which reduces the store’s revenue.
When stores have taken countermeasures, it increased magazine sales by 20%.
Reading without buying can also reduce the revenue of publishers and authors.
This behavior is considered stealing information from the magazine, and is as bad as shoplifting.
**[Belief agreement Decrease]**
Humans don’t have free will.
Everything in our universe including us is governed by the law of nature.
Our consciousness and actions are causally and inescapably determined from past states.
It is impossible to interfere with that chain of cause and effect.
Looking at things logically, the existence of free will must be denied.
**[Belief agreement Increase]**
Sugar is bad for your health.
Eating too much sugar causes dental decay.
Bacteria convert sugar to acid, which decays teeth.
Research in the UK has shown that the risk of dental decay is 1.5 times greater in people who eat a lot of sugar.
The Ministry of Health has reported that reducing sugar consumption effectively reduces this risk.
**[Neutral]**
Corsica in France
is an island belonging to France, located west of the Italian Peninsula in the Mediterranean.
Corsica is 8,680 km^2^, roughly the same size as Hyogo prefecture.
It is called Corsica in Italian, and Corse in French.
It is know as the place where the French emperor Napoleon was born.

One complete message consisted of five statements that were presented as a block. The first statement expressed the desired conclusion, and the subsequent statements comprised the persuasive argument by providing evidence or claims that supported the first statement. Note that in Japan, the cultural norm is to refrain from using cell phones on the train.

Duration of the messages (21,440–28,900 ms) depended on the number of characters. When a persuasive message ended, an interest-rating scale was presented for 4.5 s, followed by an inter-stimulus interval jittered from 4 to 10 s (average 7 s). Six persuasive messages that advocated the same conclusion (statement 1) but used different supporting statements (statements 2–5) were presented for each of the four conditions in order to intensify the persuasion. To avoid confounding the effects due to persuasion direction (increase/decrease) with those inherent to the norms/beliefs that served as the target of persuasion, we balanced norms and beliefs in each condition across participants as follows. As stated above, we chose 2 of the 50 norms, Norm_Target1/Target2, as the targets for persuasion. Half of participants were shown persuasive messages designed to increase their agreement toward Norm_Target1 and decrease their agreement toward Norm_Target2, while the reverse was true for the other half of the participants. This balanced the allocation of Norm_Target1/Target2 to NI and ND conditions across subjects. Similarly, we chose two of the fifty beliefs Belief_Target1/Target2 and balanced their allocation to BI and BD conditions. In addition to the four persuasion conditions, we also prepared a lower control condition in which we presented six blocks of neutral, non-persuasive messages (Neutral). For this condition, we prepared pure trivia on two themes (‘Corsica’ and ‘Galileo Galilei’), and presented half of the subjects with one set and the other half with the other set. For each participant, the fixed trivia theme was presented in the first statement (e.g. Corsica) and the actual trivia presented in the subsequent four statements were varied across blocks. Time course of the Neutral condition was the same as the persuasion conditions. In total, 30 blocks of messages (6 blocks × 5 conditions [ND, NI, BD, BI, and Neutral]) were presented pseudo-randomly in two sessions separated by a short break (~1 min) in the middle.

After completing all the tasks, participants left the scanner and answered a computer-based yes/no questionnaire that asked whether they were familiar with each of the 100 norms/beliefs presented in the attitude-rating tasks. Based on this question, we identified a subgroup of participants (n = 18) who were familiar with the norms that were targeted in the persuasion. When we conducted brain-behavior correlation analysis, correlation was assessed within this subgroup of participants in order to specify the neural correlates for changing ‘existing’ norms (see below for more details). Then, they were asked whether they had found anything unnatural or suspicious during the experiment. All participants answered “no” to this question. Participants were debriefed after the experiment.

*Stimulus preparation*: In the persuasion task, we balanced linguistic parameters of messages across all five conditions. These parameters included the number of characters, number of words, familiarity/frequency/imageability of words, and postpositions/parts of speech/thematic roles included in sentences. The linguistic parameters were coded using the following databases and software: WinCha 2000 R2 (Nara Institute of Science and Technology Faculty of Information Science Computational Linguistics Laboratory; Nara, Japan), NTT Database Series “Nihongo-no Goitokusei”: Lexical Properties of Japanese (SANSEIDO; Tokyo, Japan), Japanese Verb Thesaurus (Graduate School of Natural Science and Technology, Okayama University Takeuchi Laboratory; Okayama, Japan http://cl.cs.okayama-u.ac.jp/rsc/data/), and JUMAN/KNP (Department of Intelligence Science and Technology, Graduate School of Informatics, Kyoto University; Kyoto, Japan). For each linguistic parameter and their sub-categories, we confirmed that there was no statistically significant difference across conditions by conducting one-way ANOVAs.

Furthermore, we balanced perceived persuasiveness of messages across the four persuasion conditions by conducting a preliminary experiment with 14 different students (6 females and 8 males; mean age, 19.79 ± 1.37 years; range, 18–22 years). Participants were asked to rate perceived persuasiveness of the messages using an eight-point scale [1 = not at all persuasive, 8 = very persuasive]. Forty-eight persuasive messages (6 blocks × 4 conditions × 2 directions of persuasion) were presented. The persuasiveness ratings in each condition were: ND = 5.57 ± 0.30, NI = 5.00 ± 0.32, BD = 5.37 ± 0.26, and BI = 5.04 ± 0.35. We tested whether the persuasiveness of messages differed across conditions by conducting a 2 (persuasion topic: norm vs. belief) × 2 (persuasion direction: increase vs. decrease) ANOVA. Neither the main effects nor their interaction was significant.

#### Control experiment

*Participants*: Twenty-one different right-handed healthy volunteers were recruited from the same participant pool as the persuasion experiment. Although post-scan questions revealed that no participant found anything unnatural or suspicious during the experiment, during the debriefing process one participant insisted that she had noticed the bogus nature of our ‘lie-detector’ in the attitude-rating task. Thus, her data were excluded from the analysis because her ratings might have been contaminated by the desire for social desirability. Consequently, data from the remaining 20 participants were analyzed (7 females and 13 males; age: 20.75 ± 1.62 years, range: 19–25 years).

*Procedures*: The task protocol was the same as in the persuasion experiment, except that none of the messages contained any persuasive content (i.e., all 5 conditions were Neutral). As in the persuasion experiment, participants completed the persuasion and attitude-rating tasks while they were scanned in the MRI.

### Behavioral data analysis

For each participant in the persuasion and control experiment, the degree of attitude change was calculated by subtracting the 1^st^ rating from the 2^nd^ rating for each of the norms and beliefs. Statistical analyses of behavioral data were performed using R software (www.r-project.org).

To see whether messages in the persuasion experiment succeeded in moving attitudes for Norm_Target1/Target2 and Belief_Target1/Target2 in the desired directions (beyond the background attitude fluctuations these norm/beliefs showed in the control experiment), we pooled data from both the control/persuasion experiments and conducted a linear mixed model analysis that regressed the degree of attitude change on factors coding application of positive (NI and BI) and negative (ND and BD) persuasion. Additionally, other potential confounding factors common to both experiments were included as the independent variable to regress out the effect of those factors. This analysis was conducted by using the *lmer* function implemented in package *lme4* in R. Norms and beliefs were analyzed separately. For norms, the data set was constructed by collecting each participant’s data for Norm_Target1 and Norm_Target2 (thus, two data points per participant). These two norms were assigned to either NI or ND condition in the persuasion experiment, while neither of them underwent persuasion in the control experiment. Based on this data set, we regressed the degree that attitudes towards Norm_Target1/Target2 changed on the following four independent variables. (1) Whether persuasion that was intended to increase agreement was applied or not (*NI*). This factor was coded using dummy-codes (if the data belonged to the NI condition of the persuasion experiment, *NI* = 1 and if it belonged to the ND condition or control experiment, *NI* = 0). (2) Whether persuasion that was intended to decrease agreement was applied or not (*ND*) was also coded by the same rule. (3) The *initial attitude* ratings toward the targeted norm. (4) The *familiarity* with the targeted norm was coded using dummy-codes (familiar = 1, not familiar = 0). A program error precluded obtaining familiarity data from two participants in the persuasion experiment, and we excluded these participants from the analysis. As we sampled two data points from each participant, data from the same participant were not independent. In order to statistically adjust for the effect of these repeated measures, participant ID numbers were used as a random effect that affected the intercept. A similar procedure was used to analyze beliefs. Because we predicted that persuasion in NI and BI condition would increase agreement (i.e., the *β* weight of *NI* and *BI* would be greater than 0) and that persuasion in ND and BD condition would decrease agreement (i.e., the *β* weight of *ND* and *BD* would be less than 0), we used one-tailed tests for testing the significance of these factors. Similarly, we predicted that because of the ‘regression to the mean effect’, the higher the initial attitude rating, the more likely it would decrease at the second measurement, and vice versa. Thus we predicted that the *β* weight of *initial attitude* would be less than zero, and used a one-tailed test. In contrast, as we did not have any prediction regarding the effect of *familiarity*, we used a two-tailed test for this factor.

### Functional MRI Image acquisition

Functional imaging was conducted using a 3 T Trio A Tim MRI scanner (Siemens) and gradient echo T2*-weighted echo-planner images (EPI) were acquired with blood oxygenation level-dependent contrasts. Forty-two contiguously interleaved transverse EPI-image slices were acquired in each volume (slice thickness, 3 mm; no gap; repetition time, 2,500 ms; echo time, 25 ms; flip angle, 80°; field of view, 192 mm^2^; matrix, 64 × 64). Slice orientation was tilted −30° from the AC-PC line. For each subject, data were acquired in four scanning sessions (two for the persuasion task and two for the attitude rating task). For the persuasion task, 237 volumes (plus three “dummy” volumes) were acquired per session. Dummy volumes were included to allow for the T1 equilibrium effect. Similarly, 383 volumes (plus two dummy volumes) were acquired in the attitude-rating task sessions. Dummy volumes were discarded without analysis. A high-resolution anatomical T1-weighted image was also acquired for each subject. The following preprocessing procedures were performed using Statistical Parametric Mapping (SPM8) software (Wellcome Department of Imaging Neuroscience, London, UK) implemented in MATLAB R2010a (MathWorks, Natick, MA, USA) for whole-brain analysis: correction for head motion, adjustment of acquisition timing across slices, coregistration to an anatomical image, spatial normalization to the standard MNI template using the unified segmentation approach^37^, and smoothing using a Gaussian kernel with a full-width at half maximum of 8 mm.

### Functional MRI Data Analysis

We analyzed the data from the persuasion task to determine (1) whether persuasion targeting norms recruits specific neural substrates over and beyond what is recruited by persuasion in general, and (2) whether specific neural substrates are recruited depending on the direction of persuasion. Then, we jointly analyzed data from the persuasion and attitude-rating tasks to assess (3) whether brain regions that represent attitudes for norms are involved in the process by which attitudes change via persuasion.

In analyzing the data from the persuasion task, we adopted a conventional two-level approach using SPM8. A set of regressors was generated by convolving a canonical hemodynamic response function provided by SPM8 with a series of epochs. For each condition (ND, NI, BD, BI, and Neutral), the period from the start to the end of message presentation was modeled as the regressor of interest. Similarly, the rating period was modeled as regressor of no interest. The model also included session constants and motion parameters as regressors of no interest. These regressors of no interest were not included in contrasts for statistical inference. A voxel-by-voxel multiple regression analysis of these regressors was applied to the preprocessed images for each participant. Statistical inference on contrasts of parameter estimates was then performed at the second-level between-participants analysis, using a one-sample t-test. Unless otherwise stated, the statistical threshold was set at an uncorrected *p* < 0.001 at the voxel level and at *p* < 0.05, family-wise error (FWE) corrected, at the cluster level, assuming the whole brain as the search volume^38^.

We performed several contrast analyses. First, the brain regions commonly recruited across the four types of persuasion conditions were evaluated with a conjunction analysis between the four contrasts (ND-Neutral), (NI-Neutral), (BD-Neutral), and (BI-Neutral). Then, the brain regions specifically recruited during norm-directed persuasion were evaluated by testing the main effect of persuasion topic (norm vs. belief) using the contrast (ND + NI) − (BD + BI).

Second, to extract regions specifically recruited in the ND condition, an interaction contrast (ND − NI) − (BD − BI) was tested. The brain regions specific to the NI condition were evaluated by testing the contrast (NI − ND) − (BI − BD). To confirm its contribution to persuasion, we assessed the correlation between the degree of attitude change in the ND condition and the magnitude of activity in the left middle temporal gyrus (MTG), which was found to be specifically activated in the ND condition (see Results). We conducted two variations of this correlation analysis to assess whether the left MTG contributes specifically to changing ‘existing’ norms. One variation included all participants, and the other included only participants who were familiar with the norms that served as the target of persuasion (as determined by a post-scan questionnaire). To assess the magnitude of ND-specific activity in the left MTG, we extracted the parameter estimates for each condition from the first peak in the left MTG and calculated the magnitude of the interaction effect (βND − βNI) − (βBD − βBI). This provides the magnitude of activity in the ND condition that was over and above that induced by general persuasion (common effect in all conditions) and all norm-targeted persuasion (common effects in ND and NI). We further explored this brain-behavior correlation at the whole-brain level. Using a 2nd-level random-effects multiple regression analysis, we related the degree of attitude changes in the ND condition to the BOLD activation specific to the ND condition (i.e., contrast estimates for (ND − NI) − (BD − BI): these contrast estimates were derived from the same 1st-lv GLM used for other one-sample t-tests described above). For this analysis, we included only participants who were familiar with the norms that served as the target of persuasion because they had shown significant brain-behavior correlation in the left MTG.

Finally, we assessed whether brain regions that represented the attitudes for norms were involved in the process by which attitudes were changed via persuasion. First, we determined the regions that represent attitudes toward norms by conducting a parametric modulation analysis implemented in SPM8 that yielded the regions whose activity correlated more with attitude ratings for norms than with those for beliefs (positively or negatively). For this analysis, data from the pre- and post-persuasion *attitude-rating task* were combined. The stimulus presentation periods for 50 norms, including both Norm_Target1/Target2 and Norm1–48, were modeled as a regressor of interest (Attitude_Norm), and was modulated by the ratings for each argument (parametric_Attitude_Norm). Similarly, the stimulus presentation periods for 50 beliefs, including both Belief_Target1/Target2 and Belief1–48, were modeled as a regressor of interest (Attitude_Belief), and was modulated by the ratings for each argument (parametric_Attitude_Belief). Session constants, motion parameters, and response times for ratings were also included in the model as regressors of no interest. A voxel-by-voxel multiple regression analysis of these regressors was applied to the preprocessed image for each participant. Statistical inference on contrasts of parameter estimates was then performed at the second-level between-participants analysis, using a one-sample t-test. To determine if any brain regions exhibited activity that positively correlated with agreement toward norms, we performed the contrast parametric_Attitude_Norm – parametric_Attitude_Belief. Similarly, to determine if any brain regions exhibited activity that negatively correlated with agreement toward norms, we performed the contrast (−parametric_Attitude_Norm) − (−parametric_Attitude_Belief). We found that activity in left SMG correlated negatively with attitude ratings for norms (see Results). Thus, higher activity in this region was associated with less agreement with norms.

Importantly, the persuasion-task analysis indicated that in the ND condition, left SMG activity during persuasion positively correlated with the degree of subsequent attitude change (i.e., decreased agreement). Therefore, we assessed whether or not this left SMG region overlapped with the left SMG region that was indicated by the attitude-rating task analysis to code for decreased agreement with norms. First we defined a 5-mm spherical region centered at the first peak in the left SMG as determined by the analysis of the attitude-rating tasks. Then, we assessed whether significant activity related to attitude-change was found in this search volume during the ND condition by conducting small-volume correction analysis with a significance level of FWE *p* < 0.05 for magnitude of activation (initial height threshold: *p* < 0.001). Here, we looked for a correlation between attitude-change and brain activity by using the fMRI data from the persuasion task. The relevant voxels were selected by analyzing independent fMRI data from the attitude-rating task. Hence, our analysis effectively prevented circularity.

The datasets generated during and/or analysed during the current study are available from the corresponding author on reasonable request.

## Results

### Behavioral Results

#### Baseline agreement toward norms (beliefs) before persuasion

First, we assessed the degree of baseline (pre-persuasion) agreement toward norms and beliefs in the first attitude-rating task. For each participant, this was calculated by averaging ratings for 50 norms and 50 beliefs that were presented. In the control experiment, mean agreement across participants were: norms 6.348 ± 0.221, beliefs 5.871 ± 0.098. Additionally, these values in the persuasion experiment were: norms 6.684 ± 0.129, beliefs 5.913 ± 0.088.

#### Persuasion-irrelevant background attitude changes

Next, to precisely interpret the degree of persuasion-induced attitude change, we assessed persuasion-irrelevant background attitude change. To do so, we examined the changes in attitudes toward Norm1–48 and Belief1–48 because these norms/beliefs did not undergo persuasion. Each participant’s attitude change toward these 48 norms and 48 beliefs was averaged separately. As we obtained this data twice (once in the control experiment and once in the persuasion experiment), and because the degree of attitude change did not differ between these experiments (norms: *p* = 0.246, beliefs: *p* = 0.358), we combined the data. The average change across participants for norms and beliefs were: norms 0.141 ± 0.041, beliefs 0.029 ± 0.039. The results of the t-tests showed that agreement towards norms increased significantly (*p* = 0.001), whereas that toward beliefs did not change significantly (*p* = 0.452). Consequently, when directly compared, norms showed greater positive attitude change than beliefs (*p* = 0.037). Furthermore, because repeated measurement of attitudes can affect results (‘regression to the mean effect’), we confirmed that this difference was evident even after controlling for the initial attitude. We conducted a multiple regression analysis in which two factors coding (1) category (dummy codes: norm = 1, belief = 0) and (2) initial attitude ratings were entered simultaneously as predictors of the degree of attitude change. *β*s for both factors were significant (category: *β* = 0.341, *p* = 1.370 × 10^−15^; initial attitude: *β* = −0.324, *p* = 2.000 × 10^−16^).

In addition to assessing attitude changes for Norm1–48 and Belief1–48, we also examined attitude changes for Norm_Target1/2 and Belief_Target1/2 in the *control* experiment because these norms/beliefs did not undergo persuasion in that experiment. This provides important information about the background attitude fluctuation for the actual norms/beliefs that were targeted in the persuasion experiment. The average changes across participants for these norms and beliefs were: 1.025 ± 0.359 for Norm_Target1/2, and 0.225 ± 0.344 for Belief_Target1/2. While attitudes towards Norm_Target1/Target2 showed significant positive change (*p* = 0.007), those towards Belief_Target1/Target2 did not show any significant change (*p* = 0.517). Consequently, directly testing whether Norm_Target1/Target2 showed greater positive attitude changes than did Belief_Target1/Target2 revealed a marginally significant difference (*p* = 0.058, one-tailed).

The tendency for greater agreement toward norms but not toward beliefs could result from a priming effect. In the first attitude-rating task, participants were repeatedly exposed to sentences referring to different norms, which might have elevated their normative consciousness. This interpretation is consistent with findings that priming normative concepts (e.g., benevolence, equality, conformity etc.) by showing texts related to these norms increases subsequent compliance with them^39–41^. In the present study, this effect might have transcended specific norms and elevated agreement toward norms in general.

#### Persuasion-induced attitude changes

**Figure 2:**
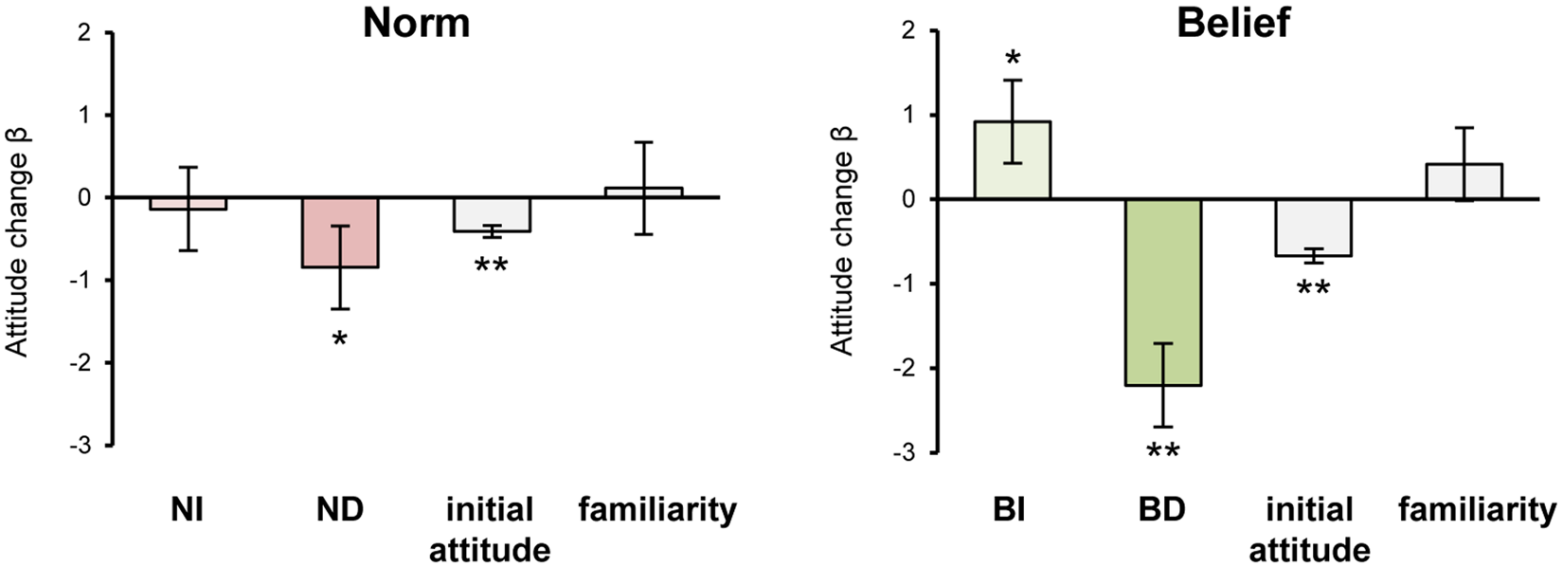
Behavioral Results. *Left*, The effects (*β* weights) on the degree of attitude change for positive (NI) and negative (ND) persuasion, initial attitudes, and familiarity to targeted norms. *Right*, The effects on the degree of attitude change for positive (BI) and negative (BD) persuasion, initial attitudes, and familiarity to targeted beliefs. Error bars depict SEM; ^+^ *p* < 0.1, *p < 0.05, **p < 0.01.

Finally, we tested whether the persuasion task successfully changed attitudes beyond what could be expected from natural fluctuation. This was done by conducting a linear mixed model analysis that directly compared the attitude changes for the same norms/beliefs (i.e., Norm_Target1/Target2 and Belief_Target1/Target2) in the persuasion and control experiments. The results summarized in Fig. 2 shows that all persuasion conditions but NI significantly moved participant attitude in the intended direction (ND: *β* = −0.847, *p* = 0.049, one-tailed; BI: *β* = 0.919, *p* = 0.033, one-tailed; BD: *β* = −2.202, *p* = 1.195 × 10^−5^, one-tailed).

The above linear mixed model analysis also revealed that *βs* for the initial attitude were significantly negative for both norms (*β* = −0.411, *p* = 9.400 × 10^−8^, one-tailed) and beliefs (*β* = −0.670, *p* = 4.170 × 10^−12^, one-tailed), meaning that the higher the initial rating, the more likely it decreased in the second test, and vice versa. This effect was replicated in another linear mixed model analysis we ran to explore factors that influenced variation of attitude changes in the persuasion experiment. In that analysis, predictors of the degree of attitude change were (1) initial attitudes, (2) familiarity to targeted norms/beliefs, and (3) degree of interest in the persuasive messages, a persuasion experiment specific factor. The results replicated the effect of initial attitudes, and showed that greater interest in persuasive messages led to more successful persuasion (see Supplementary Results). We confirmed that our fMRI results were not confounded by these factors by conducting additional analyses in which the effect of these factors was regressed out (see Supplementary Results; Supplementary Figure 2).

### fMRI Results

#### Brain regions processing persuasive messages

**Figure 3:**
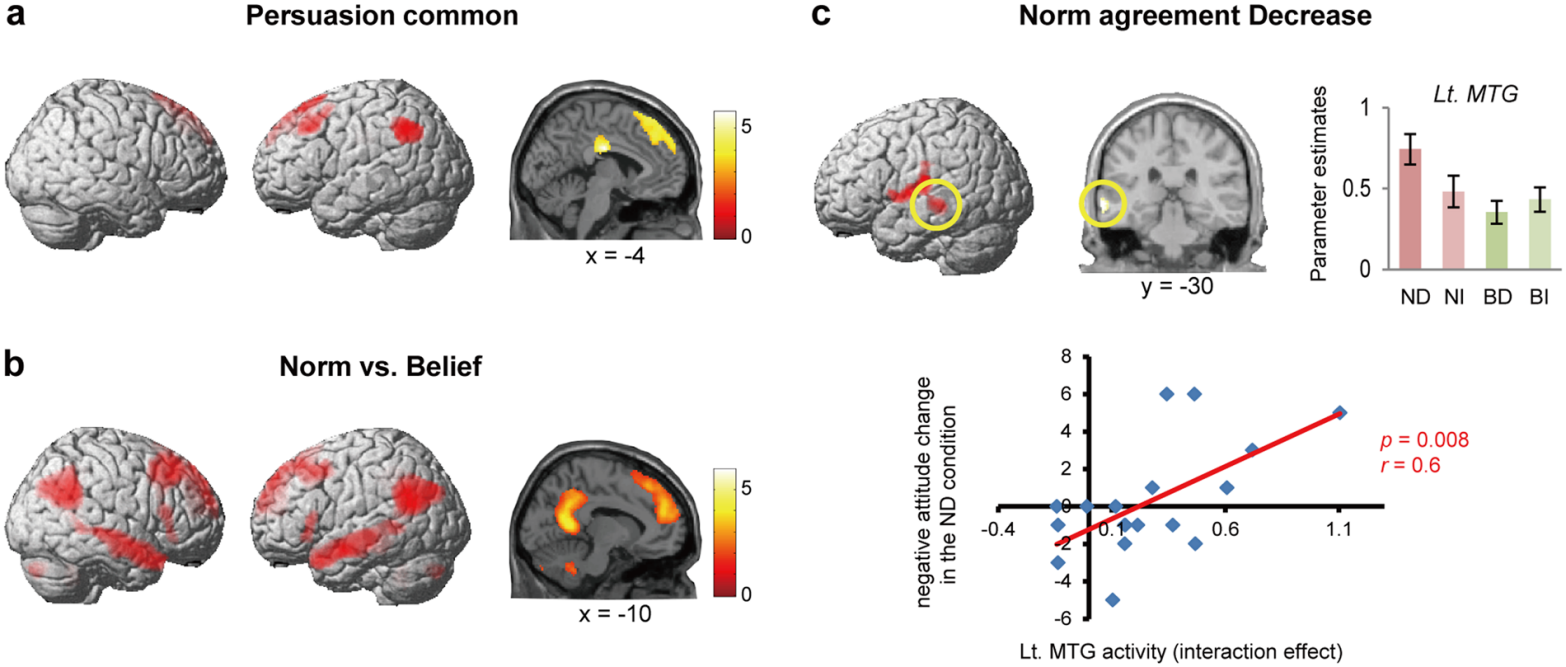
Brain regions activated during the persuasion task. (**a**) Brain regions commonly activated by all conditions. (**b**) Brain regions activated by norm-directed persuasion compared with those activated by belief-directed persuasion (direction non-specific in both cases). (**c**) Left middle temporal gyrus was specifically recruited in persuasion that designed to *decrease* agreement with norms. The activity found by the contrast (ND − NI) − (BD − BI) (*upper panel*) positively correlated with the degree of persuasion-induced attitude change across participants (*lower right*). Activation maps in (**a**–**c**) have statistical thresholds of *p* < 0.001, corrected to *p* < 0.05 for multiple comparisons using cluster size, assuming the whole brain as the search volume. Error bars depict SEM. *ND*, norm-agreement decrease. *NI*, norm-agreement increase. *BD*, belief-agreement decrease. *BI*, belief-agreement increase.

First, we looked for brain regions that play a general role in persuasion by searching for regions commonly recruited across the four conditions. A conjunction analysis between the four contrasts (ND-Neutral), (NI-Neutral), (BD-Neutral), and (BI-Neutral) showed that regardless of their targets or the direction, reading messages intended to persuade recruited regions in the left part of the dLPFC and dMPFC that have been implicated in other persuasion studies (Fig. 3a and Table 2)^24–26^.

#### Brain regions processing persuasive messages targeting social norms

Next, we determined whether any brain regions were recruited more when persuasion targeted norms than when it targeted non-normative issues. Persuasive messages directed towards norms activated a set of brain regions including the bilateral temporal pole, TPJ, and the left dMPFC (Fig. 3b and Table 2). These regions were revealed through the contrast (ND + NI) − (BD + BI), which shows that they engage in processing norm-targeting persuasive messages, irrespective of the direction of persuasion. This result was replicated even when the difference in semantic content (i.e., social factor) between norm- and belief-targeted messages was factored in (see Supplementary Results).

**Table 2:** Clusters of activation in the persuasion task.

Contrast	Cluster						
	Region	§	Size	t-value	x	y	z
***Persuasion common [(ND-Neutral)*** ∩ ***(NI-Neutral)*** ∩ ***(BD-Neutral)*** ∩ ***(BI-Neutral)]***							
	cingulate gyrus	302	320	5.74	−4	−16	28
	lt. supramarginal gyrus		803	5.15	−58	−48	42
				5.03	−50	−54	40
	lt. superior frontal gyrus		1027	4.92	−10	30	60
				4.47	−6	44	50
				4.31	−6	36	42
	lt. middle frontal gyrus		409	4.51	−44	24	44
				4.09	−42	32	42
				3.64	−38	12	58
***Norm vs. Belief [(ND*** + ***NI)*** − ***(BD*** + ***BI)]***							
	lt. supramarginal/angular gyrus	303	2810	11.9	−38	−56	22
				11.2	−42	−78	32
				9.13	−46	−56	30
	lt. parahippocampul gyrus		3650	10.28	−28	−34	−14
	lt. middle temporal gyrus			9.42	−54	2	−20
	lt. superior temporal sulcus			9.31	−54	−18	−8
	lt. posterior cingulate cortex/precuneus		4685	10.11	−4	−58	16
				7.85	−8	−52	32
	rt. posterior cingulate cortex			7.81	14	−54	18
	rt. supramarginal/angular gyrus		1914	8.77	44	−76	34
				8.28	46	−48	26
				8.23	60	−54	34
	rt. middle temporal gyrus		2832	8.04	58	4	−20
	rt. superior temporal sulcus			7.88	50	−6	−14
				7.32	48	−18	−6
	lt. superior frontal gyrus		7968	7.3	−10	48	26
	rt. middle frontal gyrus			7.07	24	28	40
	lt. middle frontal gyrus			7.02	−24	24	46
	rt. cerebellum		323	6.1	20	−82	−36
	rt. cerebellum		508	5.97	6	−52	−38
	cerebellar tonsil			5.02	0	−56	−44
	lt. cerebellum			4.97	−12	−48	−38
	lt. cerebellum		315	5.21	−16	−84	−34
				4.75	−22	−74	−32
	rt. inferior frontal gyrus		397	5.07	54	24	12
				4.45	46	28	−4
	lt. inferior frontal gyrus		328	5	−48	24	−4
				4.85	−52	22	4
***ND specific [(ND-NI)*** − ***(BD-BI)]***							
	lt. middle temporal gyrus	263	671	4.82	−68	−30	−4
	lt. superior temporal gyrus			4.64	−56	−8	8
	lt. postcentral gyrus			4.38	−66	−20	14
***NI specific [(NI-ND*** *)* − ***(BI-BD)]***							
	n.s.						

Significance level: *p* < 0.001 with cluster correction for multiple comparisons (*p* < 0.05). ^§^Minimum cluster size for corrected significance of *p* < 0.05. Size: Number of voxels. *t*-value: maximum *t*-values at the peak voxels. x, y, z: Montreal Neurological Institute (MNI) coordinates of the peak voxel. rt.: right, lt.: left.

#### Brain regions activated when being persuaded to agree less with norms

Next, we assessed whether recruited brain regions depended on the direction of persuasion. In addition to the non-specific norm-persuasion regions, we also found that different neural substrates were recruited depending on the direction of persuasion directed toward norms. The interaction contrast (ND − NI) − (BD − BI) determined that the left middle temporal gyrus (MTG) was specifically activated in the ND condition (Fig. 3c and Table 2). Indeed, this region’s activity was positively correlated with the degree of the persuasion effect (i.e., persuasion-induced *negative* attitude changes), indicating that this region is truly involved in the attitude changes observed in the ND condition (*p* = 0.008, *r = *0.6). Tellingly, this relationship between brain activity and attitude change was only observed when participants who were familiar with the targeted norms were included in the analysis (n = 18). This suggests that left MTG plays an important role in changing attitudes toward established norms through persuasion.

**Figure 4:**
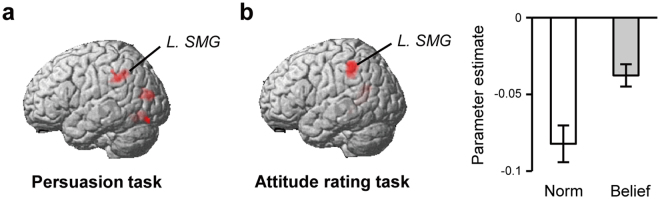
The left SMG was involved with the persuasion process as well as with representation of attitudes (less agreement) regarding norms. (**a**) Magnitude of left SMG activity during persuasion (Norm-agreement-Decrease condition) was positively correlated with the degree of attitude-change (i.e., decrease of agreement) across participants. (**b**) Left SMG activity negatively correlated with the degree of agreement toward norms in the attitude rating tasks (parametric modulation). In (**a**,**b**) the statistical threshold is *p* < 0.001, corrected to *p* < 0.05 for multiple comparisons using cluster size, assuming the whole brain as the search volume. *SMG*, supramarginal gyrus.

We further explored whether any other brain regions showed a similar relationship with the persuasion effect by conducting a whole-brain correlation analysis between activity during the ND condition and the degree of successful ND persuasion for participants who were familiar with the targeted norms. This was done to capture regions whose activity was related to attitude change, but due to the large variance across participants, was too low to be captured by the standard categorical analysis that averages effects across participants. We found a positive correlation in the left supramarginal gyrus (SMG) (Fig. 4a and Table 3). With a rather lenient threshold (thresholded at *p* < 0.005, cluster corrected at *p* < 0.05), the correlation in the left MTG was also replicated. Additional analyses confirmed that the correlations between the persuasion effect and brain activity in the left SMG/MTG was specific to the ND condition, rather than merely reflecting persuasion effect-related activity that was common to other conditions (see Supplementary Results).

**Table 3:** Whole-brain correlation analysis (brain regions whose activity correlated with the degree of attitude change in the ND condition).

Contrast	Cluster						
	Region	§	Size	t-value	x	y	z
	lt. cuneus	151	216	6.79	−24	−78	14
	lt. supramarginal gyrus		410	6.71	−26	−48	40
				4.9	−44	−44	32
				4.56	−38	−32	32
	rt. inferior temporal gyrus		187	6.66	42	−50	−26
				5.88	54	−46	−20
				5.84	50	−40	−12
	lt. cerebellum		324	5.01	−14	−66	−16
				4.6	−16	−72	−10
				4.48	−10	−60	−10

Significance level: *p* < 0.001 with cluster correction for multiple comparisons (*p* < 0.05). ^§^Minimum cluster size for corrected significance of *p* < 0.05. Size: Number of voxels. *t*-value: maximum *t*-values at the peak voxels. x, y, z: Montreal Neurological Institute (MNI) coordinates of the peak voxel. rt.: right, lt.: left.

Interestingly, no specific activation was found related to shifting attitudes toward norms (NI condition, contrast: [NI − ND] − [BI − BD]). Results from other contrasts are shown in Supplementary Table 1.

#### SMG activity was associated with less agreement with norms

Finally, we assessed whether brain regions that represent attitudes for norms are involved in the process through which attitudes change via persuasion. We found that left SMG represented attitude toward norms and also tracked the persuasion-induced changes in attitudes, providing evidence for the neural link between the representation of attitudes and their changes through persuasion.

To find brain regions that specifically code attitudes regarding norms, we analyzed fMRI data obtained during the attitude-rating tasks. Practically, we searched for brain regions whose activity correlated more with attitudes for norms than with those for beliefs (positively or negatively). Although we did not find a significant result for positive correlations, the contrast (−parametric_Attitude_Norm) − (−parametric_Attitude_Belief) revealed that activity in the left SMG correlated negatively with attitude ratings for norms. Thus, higher activity in this region was associated with less agreement with norms (Fig. 4b and Table 4). To make sure that the left SMG activity was associated specifically with decreased agreement toward norms, but not with increased agreement toward beliefs (which is the alternative interpretation of the contrast [−parametric_Attitude_Norm] − [−parametric_Attitude_Belief]), we conducted additional confirmatory analyses that employed the contrast (−parametric_Attitude_Norm) as an inclusive mask, or parametric_Attitude_Belief as an exclusive mask. These analyses confirmed that the left SMG activity was specifically associated with less agreement toward norms (Supplementary Figure 1).

**Table 4:** Clusters of activation in the attitude-rating task.

Contrast	Cluster						
	Region	§	Size	t-value	x	y	z
***Norm specific negative correlation [***(−***parametric_Attitude_Norm)*** − (−***parametric_Attitude_Belief)]***							
	lt. posterior cingulate cortex	229	536	6.22	−16	−56	22
				4.32	−12	−50	8
				3.98	−10	−46	0
	lt. supramarginal gyrus		481	4.81	−42	−40	38
				4.17	−48	−42	50
	rt. postcentral gyrus		387	4.22	62	−14	34
	rt. supramarginal gyrus			4.14	42	−38	40
				3.98	48	−34	44
***Norm specific positive correlation [(parametric_Attitude_Norm*** − ***parametric_Attitude_Belief)]***							
	n.s.						

Significance level: *p* < 0.001 with cluster correction for multiple comparisons (*p* < 0.05). ^§^Minimum cluster size for corrected significance of *p* < 0.05. Size: Number of voxels. *t*-value: maximum *t*-values at the peak voxels. x, y, z: Montreal Neurological Institute (MNI) coordinates of the peak voxel. rt.: right, lt.: left.

Importantly, the persuasion-task analysis had indicated that in the ND condition, left SMG activity during persuasion positively correlated with the degree of subsequent attitude change (i.e., decreased agreement). Therefore, we assessed whether or not this left SMG region overlapped with the left SMG region that was indicated by the attitude-rating tasks to code for less agreement with norms. First we defined a 5-mm spherical region centered at the first peak in the left SMG as determined by the analysis of the attitude-rating tasks. Then, we assessed whether significant activity related to attitude-change was found in this search volume during the ND condition of the persuasion task by conducting small-volume correction analysis with a significance level of FWE *p* < 0.05 for magnitude of activation (initial height threshold: *p* < 0.001). This analysis yielded a significant result, suggesting that the left SMG regions activated during the persuasion task and the attitude-rating tasks actually overlapped. This significant result was replicated when we defined search volume based on the results of the initial analysis of the attitude-rating task that used inclusive/exclusive mask (Supplementary Figure 1), and applied this search volume to a small-volume correction analysis. Thus, a plausible account of the data is that as persuasion convinced participants to agree less with a norm, left SMG activity that reflected this lack of agreement increased. This idea is supported by previous reports showing that left SMG responds to the intentional violation of norms, especially when the violation was justified^42,43^.

## Discussion

Norms regulate human social behavior and have a deep relationship with important social phenomena such as cooperation, collective action, and social order. Thus, changes in norms have a great impact on our society, and individual’s changes in attitudes toward norms are critical to such a process. Here, we showed that changes in norm-related attitudes that occur through persuasion are mediated by multiple layers of brain networks.

First, we showed that the dLPFC and dMPFC were commonly recruited across all persuasion conditions, suggesting their general role in attitude change or belief update. This is consistent with previous persuasion studies that dealt with non-normative beliefs^24–26^. The notion that the dLPFC and dMPFC are engaged in general belief update is also supported by findings from studies that did not directly assess persuasion. For example, one study showed that the left dLPFC mediated belief update in a stock market task irrespective of whether such update was based upon private or social information^44^. Another study showed that this region mediated the effect of instructed knowledge on reward probability learning^45^. As for the dMPFC, Wittmann *et al*. showed that this region was commonly involved in the update of belief regarding one’s ability or the ability of others^46^.

Second, we showed that in addition to these general regions, persuasive messages directed toward norms activated a specific set of brain regions including the temporal pole, TPJ, and the dMPFC. Thus, in norm-persuasion, these regions may handle social information inherent to normative issues and this might help changing attitudes toward norms. This interpretation is consistent with findings that the temporal pole, TPJ, and dMPFC engage in processing a variety of social information (e.g., human interaction or the mental states of others)^47–53^, and are also used when making moral judgments that must utilize such information to make normative decisions^18,54^.

Third, we showed that beyond these regions, the left MTG was recruited specifically when persuasion was designed to decrease agreement with norms. A straight interpretation of our results is that the left MTG plays a directionally specific role in norm-persuasion, shifting one’s attitude *away* from accepted norms. This interpretation is supported by the correlation between left MTG activity during persuasion and the degree of persuasion-induced attitude change in the ND (but not NI) condition, and the fact that this was only observed in participants who were familiar with targeted norms. Left MTG may contribute to such attitude changes through roles in logical reasoning and a reappraisal process. People naturally tend to conform to norms because this tendency benefits them from an evolutionary-psychological standpoint by stabilizing society^4,55^. Thus, when people change their attitudes *away* from what they recognize as accepted norms, they absolutely require deep logical reasoning or a reappraisal process. This idea is consistent with known roles for MTG in cognitive reappraisal^56^, logical reasoning regarding deontic rules^57^, and more widely in conceptual/language processes essential for logical reasoning^58,59^. Although we prefer the above interpretation, the fact that persuasion was not successful in the NI condition keeps us from ruling out the possibility that the left MTG’s activity reflects general success in norm-persuasion, without any directional specificity. Because the perceived persuasiveness of the messages was balanced across all conditions, the lack of significant effect in the NI condition probably did not result from messages that were less persuasive. Rather, it might be partially accounted for by the significant increase in the degree of agreement observed in the post-test of the control experiment that did not use persuasion. This elevated baseline, and possibly an accompanying ceiling effect, could have hindered detection of persuasion-induced increases of agreement in the NI condition. Another possibility is that the MTG activity merely reflected increased language processing (or message reading) that was induced by the novelty of messages that went against norms or just by chance, and hence the observed correlation between MTG activity and the degree of persuasion effect would simply reflect the fact that more reading led to greater persuasion effect. However, this interpretation is unlikely. First, as mentioned above, MTG results remained the same even after the effect of message novelty (i.e., how much initial attitudes toward targeted norms differed from what was advocated in the persuasive messages) was removed (see Supplementary Results; Supplementary Figure 2). Second, as the correlation between MTG activity and the persuasion effect was only observed in the ND condition, it cannot reflect a general relationship between message reading and persuasion effect.

Finally, we showed that left SMG was also recruited in persuasion that decreased agreement with norms. In combination with data from attitude-rating tasks, we specified that left SMG activity represented one’s attitude toward norms, and tracked the persuasion-induced changes in attitudes away from agreement. Within the division of roles between left SMG and MTG, SMG directly represents the changed attitude itself and MTG reflects the reasoning process that propelled the change. In this scenario, the relationship between reasoning and attitude change is less direct and can explain why SMG activity correlated more strongly with attitude change. Our notion that the left MTG and left SMG collaborate in changing attitudes regarding norms through persuasion is also supported by their strong anatomical connection through the perisylvian pathway^60^. This pathway is particularly developed in humans, and has been suggested to be related to the evolution of verbal communication^61^.

Taken together, our findings suggest the following neural model of norm-persuasion. Regions including the dLPFC and dMPFC provide the common basis for attitude change. Further, the norm-persuasion process necessitates contribution of the following regions outside these regions. First, temporal pole, TPJ, and dMPFC process social information inherent to normative issues. Second, left MTG handles extra reasoning/reappraisal if and only if the proposed direction of attitude change is away from accepted norms. Finally, the left SMG represents attitudes toward norms and tracks the persuasion-induced changes in attitudes.

These suggested functions of each brain region/network indicate that neural substrates for norm persuasion are culturally independent. Still, how strongly they are recruited might depend on cultural background. Because the strength of norms to bind people depends on culture^8^, cultural regions with a greater aversion to breaking norms (such as Japan, where the present study sample was collected), people might have greater difficulty in decreasing agreement with them and might well require greater logical reasoning/reappraisal process. In such a situation, the left MTG that supports this process might well show greater activation during persuasion, and this could be why we were able to detect such a clear relationship between the left MTG and norm persuasion in this study. Nevertheless, future cross-cultural study is necessary to confirm these predictions.

High agreement (less agreement) with accepted norms can be seen as a kind of conformity (nonconformity). Comparing our findings with those from studies that assess the neural basis of conformity can help tease apart subtle differences between these two issues. Reports have shown conflict-related activity in the dorsal anterior cingulate, dMPFC, insula, and amygdala when participants found that their opinions/expectations contradicted with those of their peers or expert advisers, whereas reward-related activity was observed in the striatum when they found that their opinions matched those of their peers/experts^14,62–69^. These regions are mostly different from the ones we identified here with regard to persuasion of social norms. When the tasks and their elicited behaviors are examined more closely, we can see differences that are reflected in the activation of separate brain networks. The conformity studies mentioned above assessed a process in which participants changed or updated their opinions/expectations based purely on how the majority of their peers/experts think, without much reference to rationale behind it. On the other hand, in our study, participants were presented with reasons why specific norms should be accepted or discarded and changed their attitudes by considering them. Thus, the driving force behind their changes in attitude was not necessarily restricted to the desire to match their peers/experts, but required greater logical reasoning. The qualitative difference between the cognitive processes used when changing attitudes/opinions in the conformity studies and those used in our persuasion study was thus evident in the different brain networks that we observed. This difference could parallel the difference between how people change their beliefs in ‘descriptive’ and ‘injunctive’ norms^70,71^; whereas the former could change through the similar neural mechanisms clarified by previous conformity studies, the latter could do so through neural mechanisms clarified in the present study. Of note, the dMPFC has been identified in both previous conformity studies and our current norm-persuasion study. This overlap can be explained as updating normative beliefs (or values), which is a process that is common to both situations. This notion is consistent with Apps and Ramnani^72^, in which normative (but not subjective) value was found to be coded specifically in the anterior part of the dMPFC. They also showed that the more caudal region of the MPFC codes both normative and subjective values. In the present study, both norm-persuasion and general-persuasion regions were found in the dMPFC, although the former was located in more anteriorly. This is consistent with the Apps and Rammani findings.

In the present study, some of our analyses were carried out on a subset of participants (n = 18). Although no formal power analysis was conducted, we believe 18 participants is enough for conventional fMRI analysis^73^. The fact that we found significant results with this relatively small sample size (compared to the full 27 participants) indicates that they have been caused by large effect sizes. Nevertheless, this rather small sample size is a limitation that should be considered.

In the present study, in order to successfully change attitudes regarding norms, we targeted one norm or belief in each condition for intensive persuasion. Although behaviorally we succeeded in this goal (except for NI condition), and despite balancing the targeted norms (beliefs) across participants, the generality of our conclusions might still be in question. Given that this is the first study that has assessed neural processes underlying norm-persuasion, more research is needed to replicate and extend the results observed here. Similarly, as we measured attitude only before and after persuasion in order to minimize the possibility that participants noticed our intent, we could not track their attitude changes trial-by-trial and conduct analyses using that type of data. Future studies might constructively overcome this limitation and utilize a computational approach to gain further insight into the neural process of norm change. Finally, there are two issues we think fruitful avenues for further investigation. Fist, the present study focused on the effect of ‘message’, a primary factor in persuasion^74^, and did not assess other ones like the effect of ‘source’ (who provides the message), nor an interaction between them. Clarifying this in the context of norm-persuasion may be important. Second, it is important to assess whether different persuasion media (e.g. text message, auditory presentation, videos etc.) recruit same or different neural substrates. Although recent studies of health promotion indicate that MPFC is commonly recruited by audio-visual text-based and video-based persuasion^23,26^, evidences are still scarce, to say nothing of other brain regions we found to be norm-persuasion specific here. So, this task is left to future research.

## Conclusion

In conclusion, we successfully showed that exposure to norm-directed persuasion recruits different brain processes than general belief-directed persuasion does, and is characterized by directional specificity. These findings may contribute to ongoing studies regarding social group dynamics^75–77^, especially those assessing how norms or cultures are transmitted through communication in a social network^13,78,79^. Future studies would benefit from adopting biologically valid models that take into account the structure of brain processes. We believe that our findings can contribute to the construction of such a model for the process by which norms change and to a better understanding of how language communication shapes social structure.

## Electronic supplementary material

Supplementary Information
