# Supplementary material for: The Neural Basis of Changing Social Norms through Persuasion
Source: Sci Rep. 2017 Nov 24;7:16295. doi: 10.1038/s41598-017-16572-2 (PMC5701130; doi:10.1038/s41598-017-16572-2)
Supplement: Supplementary file 1 — Supplementary Information [file 41598_2017_16572_MOESM1_ESM.pdf]

Title: The Neural Basis of Changing Social Norms through Persuasion

Short title: The Neural Basis of Norm change

Authors:

Yukihito Yomogida [ab]*, Madoka Matsumoto[a], Ryuta Aoki[a,], Ayaka Sugiura[c,d], Adam N. Phillips[a], and Kenji Matsumoto[a]*

Author Affiliations:

[a]Brain Science Institute, Tamagawa University, Tokyo, 194-8610, Japan

[b]Department of Mental Disorder Research, National Institute of Neuroscience, National Center of Neurology and Psychiatry

[c]Japan Society for the Promotion of Science, Tokyo, 102-0083, Japan

[d]Dept Life Sci, GSAS, Univ of Tokyo, Tokyo, 153-8902, Japan

***Corresponding Authors:**

Dr. Kenji Matsumoto

Dr. Yukihito Yomogida

Tamagawa University Brain Science Institute

6-1-1 Tamagawa-gakuen, Machida, Tokyo 194-8610, Japan

Tel/Fax: +81-42-739-7231

Email: matsumot@lab.tamagawa.ac.jp, yomogita@lab.tamagawa.ac.jp

Keywords: social norm, persuasion, belief, social influence, attitude

[Supplementary Results]

<u>The main fMRI results were replicated when taking into account the confounding factors that influenced attitude-change variation in the persuasion experiment.</u>

To remove the effects of potentially confounding factors from the conditional differences in fMRI signals, we looked for factors that may have influenced how much attitudes changed in the persuasion experiment. First, we pooled the data from all participants across the four persuasion conditions (thus, four data points per participant) and conducted a linear mixed model analysis using the *lmer* function implemented in package *lme4* in R. Here, the dependent variable was the 'persuasion-effect', the degree to which participants changed their attitudes in the direction suggested by the persuasive messages, rather than the degree of raw positive/negative attitude change. This measure was taken because one of the independent variables (participants' interest in persuasive messages; see below) was predicted to influence the 'effect of persuasion' rather than simply move participants' attitude positively or negatively. To code persuasion-effect, in the ND and BD conditions we reversed the polar character of the raw attitude change values (e.g., -6 → 6, 1 → -1), because in these conditions *negative* attitude change indicates *positive* persuasion-effect and vice versa. On the other hand, in the NI and BI conditions, we simply used the raw attitude change values themselves. Subsequently, these persuasion-effects were regressed on the following three independent variables. (1) The participants' initial attitudes toward the targeted norms/beliefs (*initial attitude*), which had been measured during the first attitude-rating task (before persuasion). Because of statistical regression to the mean, we expected that in the NI and BI conditions, the more (less) participants initially agreed with a targeted norm/belief, the less (more) likely they would be to increase their levels of agreement, thus showing lesser (greater) persuasion-effects. The reverse pattern was expected in the ND and BD conditions. Therefore, for the NI and BI conditions, initial ratings for each targeted norm/belief were reversed to code the effect of initial attitudes (e.g., 8 → 1, 1 → 8), whereas the actual initial ratings were used for the ND and BD conditions. Consequently, if regression to the mean was predicted, the β weights of initial attitude should be positive. (2) The familiarity to the targeted norm/belief (*familiarity*) was coded using dummy-codes (familiar = 1, not-familiar = 0). (3) The degree of interest participants felt while reading the persuasive messages (*interest*). For this variable, an average value obtained from six blocks was used for each condition. We predicted that the β weight of this factor would be positive because greater interest would lead to a greater persuasion-effect. As we sampled four data points from each participant, data from the same participant are not independent. To statistically adjust for the effect of these repeated measures, participant ID number was used as a random effect that affected the intercept. Because a program error precluded obtaining familiarity data from two participants, we excluded these participants from the analysis. The results showed that initial attitude (β = 0.517, p = 3.345×10^-10, one-tailed) and interest (β = 0.269, p = 0.027, one-tailed) obtained statistically significant β weights, indicating that (1) initially opposing attitudes raised the level of persuasion-effects via a regression to the mean effect, and (2) greater interest led to greater persuasion-effects.

We then regressed out the effects of initial attitude and interest (the confounding factors) when testing conditional differences in fMRI signals. To analyze the main effect of persuasion topic evaluated by the contrast (ND + NI) – (BD + BI), first we assessed the net effects of confounding factors expected to be found in this contrast. For each participant, the effect of initial attitude (i.e., [$ND_{initial\ attitude}$ + $NI_{initial\ attitude}$] – [$BD_{initial\ attitude}$ + $BI_{initial\ attitude}$]) and the effect of interest (i.e., [$ND_{interest}$ + $NI_{interest}$] – [$BD_{interest}$ + $BI_{interest}$]) were calculated. These values were entered as covariates of no interest when testing the effect of contrast (ND + NI) – (BD + BI), and the second-level one-sample t-test showed essentially the same results as those from the original analysis (thresholded at $p < 0.001$, cluster corrected at $p < 0.05$) (Supplementary Figure 2a). We conducted a similar analysis for the interaction contrast (ND – NI) – (BD – BI), which explored the brain regions specific to

the ND condition. Here, the net effects of confounding factors were calculated as interaction effects of these factors (e.g., $[ND_{interest} – NI_{interest}] – [BD_{interest} – BI_{interest}]$). These values were entered as covariates of no interest when testing the contrast (ND – NI) – (BD – BI) in the second-level one-sample t-test. This analysis showed activation in left MTG similar to that obtained in the original analysis (thresholded at $p < 0.001$, cluster corrected at $p < 0.05$) (Supplementary Figure 2b). The MNI coordinate of the first peak in this cluster was exactly the same as the original one (x = -68, y = -30, z = -4) whose activity correlated with the degree of attitude change in the ND condition, indicating that the brain-behavior correlation in the left MTG was also replicated. This was further confirmed by a multiple regression analysis. When the magnitude of the left MTG activity specific to the ND condition was regressed on both the degree of attitude change and the confounding factors (initial attitude and interest), the weight of attitude change was still significant ($\beta = 0.062$, $p = 0.006$, one-tailed). Additionally, we applied a similar procedure to the whole-brain analysis that explored brain regions whose activity correlated with the degree of attitude change in the ND condition. In a second-level multiple regression analysis, the ND-specific activity (ND – NI) – (BD – BI) was covaried not only with persuasion-induced attitude change but also with the interaction effects for initial attitude and interest. This analysis showed a left SMG activation similar to that obtained from the original analysis (Supplementary Figure 2c; thresholded at $p < 0.005$, cluster corrected at $p < 0.05$). The overlap with the left SMG region that represented attitudes toward norms was also replicated with a small volume-correction analysis with a significance level of $p < 0.05$ for magnitude of activation.

In sum, all of our fMRI results as well as the brain-behavior correlations described in the main text remained after removing factors that could have influenced the degree of attitude change in the persuasion experiment.

Activity in the temporal pole, TPJ, and dMPFC does not merely reflect the social content contained in norm-targeted persuasive messages.

When we tested the main effect of persuasion topic (i.e., (ND + NI) – (BD + BI)) without any mask, we found activity in the temporal pole, TPJ, and dMPFC (Supplementary Figure 3a). Because these regions overlapped with regions known to process a wide range of social information, we worried that activity in these regions might merely reflect the difference in social content between persuasive messages that target norms and those that target beliefs. Specifically, any description of a norm explicitly or implicitly referring to people in social situations has social content, but this is not always true for beliefs. To clarify this issue and determine whether any regions responded specifically to norm-targeted messages *only* during persuasion, not merely to the social content in the messages, we employed the following exclusive masking procedure.

We focused on the fact that the difference in social content at issue also holds true for the text presented in the attitude-rating tasks, which have no persuasion intent. Therefore, we first identified brain regions that simply reflect the difference in social content between norm-related and belief-related text by contrasting brain activation elicited by reading these texts in the attitude-rating tasks. This was done by testing the contrast (Attitude_Norm – Attitude_Belief). This contrast yielded activation in the temporal pole, TPJ, and dMPFC (Supplementary Figure 3b). Then, the question is whether we are still able to find voxels that show significantly greater activation during norm-targeted persuasion (compared to belief-targeted persuasion) in the persuasion task (possibly within sub-portions of the original areas) when voxels activated by this contrast are excluded from the analysis. If so, such voxels truly reflect processing of norm-targeted messages only under the context of persuasion, rather than merely reflecting any difference in social content. To answer this question, first we thresholded the activations elicited by the contrast (Attitude_Norm – Attitude_Belief) at unc. $p < 0.05$, and then applied it as an exclusive mask in testing the norm-targeted vs. belief-targeted persuasion contrast (i.e., (ND + NI) – (BD + BI)). The threshold of this mask was set to unc. $p < 0.05$ in order to conservatively exclude voxels that merely reflect the

difference in social content. As a result, we found regions specificly related to norm-persuasion in the temporal pole, TPJ, and dMPFC (uncorrected $p < 0.001$ at the voxel level and at $p < 0.05$, family-wise error (FWE) corrected, at the cluster level; Supplementary Figure 3c), even though some voxels observed in the initial analysis had dropped out (Supplementary Figure 3d, yellow regions). This was also true when we considered the effect of the confounding factors that influenced attitude-change variation (Supplementary Figure 4). To correct for multiple comparisons in this analyses, we assumed the whole brain as the search volume rather than the smaller regions produced by exclusive masking. Thus, our results are free from any false positives due to applying masks.

Correlations between the persuasion-effect and brain activity in the left SMG/MTG is specific to the ND condition.

As noted in the Results section, a whole-brain correlation analysis revealed that the activity in the left SMG positively correlated with the degree of attitude change in the ND condition. In this analysis, our primary focus was the brain-behavior correlation in the ND condition. Thus, we used the interaction contrast (ND-NI)-(BD-BI) to search for voxels showing ND condition-specific activity, and assessed the correlation of this activity with the persuasion-effect in the ND condition. The persuasion-effect used in this analysis was not calculated as an interaction effect. Thus, whether this captured an ND condition-specific persuasion-effect relative to other conditions was not immediately clear, and we needed to check whether the correlation between the persuasion-effect and brain activity in the left SMG was specific to ND condition and not merely reflective of persuasion-effect related activity common to all conditions.

To clarify this issue, for each condition other than ND (i.e., NI, BD, BI), we assessed the correlation between the left SMG activity and the persuasion-effect. More specifically, we conducted 2nd-level random-effects multiple regression analyses similar to what we did for the ND condition. For each condition, the contrasts were selected to capture activity specific to that condition (e.g., [NI - ND] – [BI - BD] for the NI condition], and the degree of persuasion-effect in that condition was entered as the covariate of interest. To assess whether there were significant effects in the left SMG region comparable to what we had identified from the analysis of the ND condition, small volume corrections were applied to the left SMG search volume (defined by the results of the same independent attitude-rating task) with a significance level of $p < 0.05$ for the magnitude of activation (initial height threshold: $p < 0.05$ uncorrected). Even with this very lenient height threshold, we could not find significant results in any condition. These non-significant results were replicated regardless of whether analyses included (1) all the participants or (2) only participants who were familiar with the norm/belief that served as the target of persuasion in the condition of interest (as we did in the analysis of ND condition), or (3) exactly the same participants who were included in the analysis of the ND condition. Consequently, assuming that the correlation between the persuasion-effect and brain activity in the left SMG is specific to ND condition is reasonable.

Similar to the left SMG, we had found that left MTG activity positively correlated with the persuasion-effect in the ND condition. Thus, we conducted similar analyses to check whether the correlation between the persuasion-effect and brain activity in the left MTG was specific to ND condition. For each condition other than ND (i.e. NI, BD, BI), we extracted the left MTG activity specific to that condition (e.g., [βNI – βND] – [βBI – βBD] for the NI condition) and assessed its correlation with the persuasion-effect in that condition. None of the conditions showed a significant correlation comparable to what we found in the ND condition. These non-significant results were replicated regardless of the participant selection methods. Thus, we conclude that the correlation between the persuasion-effect and brain activity in the left MTG is specific to the ND condition.

Supplementary Figure 1.

Left SMG activity was specifically associated with less agreement toward norms, and was not related to greater agreement toward beliefs.

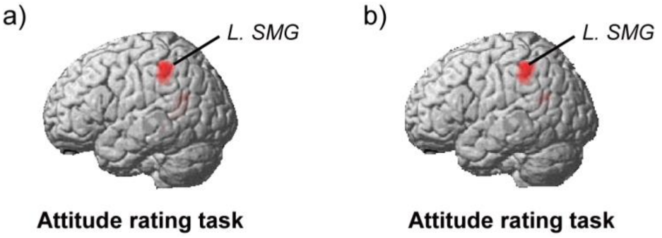

(a) Brain regions revealed by the contrast (–parametric_Attitude_Norm) – (–parametric_Attitude_Belief) inclusively masked by (−parametric_Attitude_Norm). **(b)** Brain regions revealed by the contrast (–parametric_Attitude_Norm) – (–parametric_Attitude_Belief) exclusively masked by parametric_Attitude_Belief. The statistical threshold is $p < 0.001$, corrected to $p < 0.05$ for multiple comparisons using cluster size, assuming the whole brain as the search volume.

Supplementary Figure 2.

The main fMRI results were replicated even after considering factors influencing the variation of attitude change in the persuasion experiment.

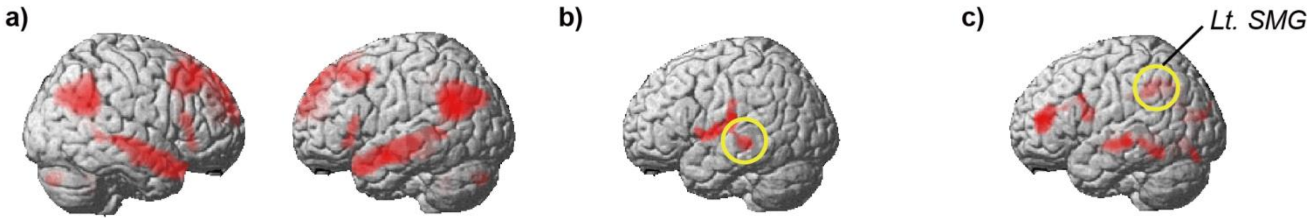

(a) Brain regions activated by norm-directed persuasion compared with those activated by belief-directed persuasion (direction non-specific in both cases) ($p < 0.001$ uncorrected, corrected to $p < 0.05$ by cluster size). **(b)** The left MTG was specifically recruited in persuasion designed to decrease agreement toward norms ($p < 0.001$ uncorrected, corrected to $p < 0.05$ by cluster size). **(c)** The magnitude of the left SMG activity during persuasion was positively correlated with the degree of persuasion-induced attitude change in the ND condition ($p < 0.005$ uncorrected, corrected to $p < 0.05$ by cluster size).

Supplementary Figure 3.

Schematic illustration of an analysis that employed exclusive masking procedure to confirm the results.

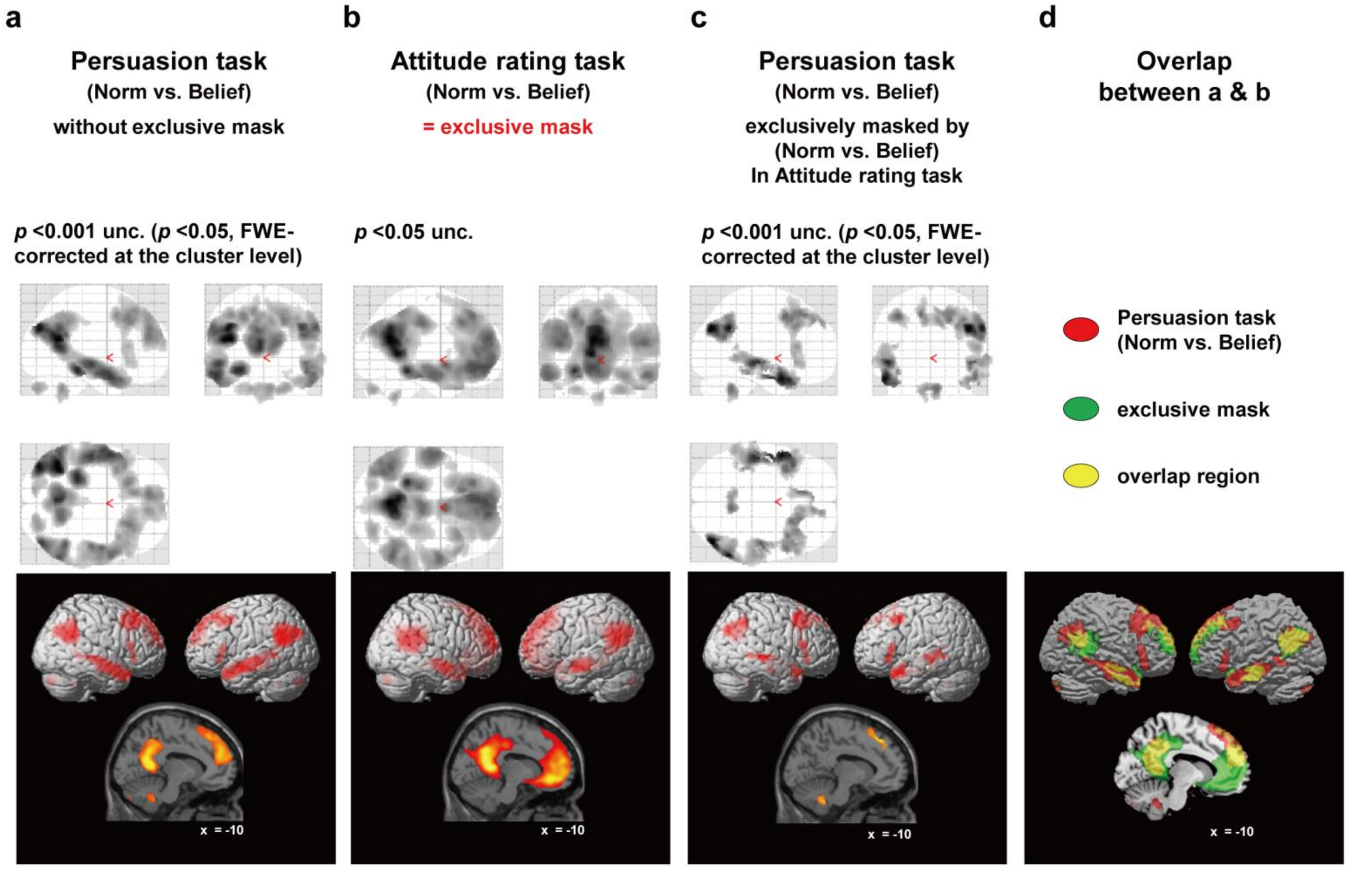

Supplementary Figure 4.

Activity in temporal pole, TPJ, and dMPFC specific for persuasion of norms, rather than a simple reflection of the social content of the messages.

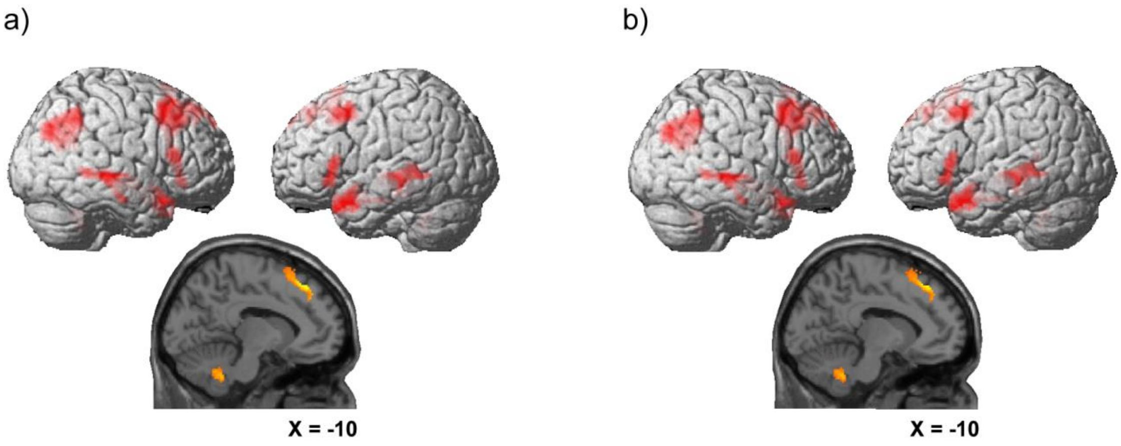

(a) Brain regions revealed by the contrast (ND + NI) – (BD + BI) exclusively masked by (Attitude_Norm – Attitude_Belief). **(b)** Brain regions revealed by the same contrast and masking procedure, with the effect of initial attitudes toward targeted norms/beliefs and the interest in persuasive messages regressed out as covariate of no interest. For the activation maps in **(a)** and **(b)**, the statistical threshold is $p < 0.001$, corrected to $p < 0.05$ for multiple comparisons using cluster size, assuming the whole brain as the search volume.

Supplementary Table 1. Clusters of activation in the persuasion task.

Contrast	Cluster Region	§	Size	t-value	x	y	z
Belief vs. Norm [(BD+BI)-(ND+NI)]							
	lt. occipital cortex	303	552	4.62	-22	-82	8
				4.38	-14	-98	18
				4.21	-22	-76	14
	rt. occipital cortex		486	4.4	38	-84	-8
				4.33	34	-86	0
				4.09	16	-96	12
Decrease vs. Increase [(ND+BD)-(NI+BI)]							
	rt. supramarginal gyrus	305	1166	6.98	50	-56	36
	rt. middle temporal gyrus		1153	6.92	60	6	-24
				5.81	64	-32	-8
				5.56	58	-18	-18
	rt. inferior frontal gyrus		478	6.77	40	44	-12
	rt. superior frontal gyrus		1229	5.58	22	32	52
				4.93	8	36	42
	rt. middle frontal gyrus			4.71	44	24	28
	lt. angular gyrus		583	5.43	-46	-60	38
	lt. middle frontal gyrus		682	5.21	-36	18	30
				4.4	-46	20	32
				4.37	-32	16	48
Increase vs. Decrease [(NI+BI)-(ND+BD)]							
	n.s.						

Significance level: $p < 0.001$ with cluster correction for multiple comparisons ($p < 0.05$).
§: minimum cluster size for corrected significance of $p < 0.05$.
Size: Number of voxels. *t*-value: maximum *t*-values at the peak voxels.
x, y, z: Montreal Neurological Institute (MNI) coordinates of the peak voxel. rt.: right, lt.: left
